# Polymer-Based Smart Drug Delivery Systems for Skin Application and Demonstration of Stimuli-Responsiveness

**DOI:** 10.3390/polym13081285

**Published:** 2021-04-15

**Authors:** Louise Van Gheluwe, Igor Chourpa, Coline Gaigne, Emilie Munnier

**Affiliations:** EA 6295 Nanomédicaments et Nanosondes, Faculté de Pharmacie, Université de Tours, 31 Avenue Monge, 37200 Tours, France; louise.vangheluwe@univ-tours.fr (L.V.G.); igor.chourpa@univ-tours.fr (I.C.); coline.gaigne15@laposte.net (C.G.)

**Keywords:** smart drug delivery systems, polymers, dermatology, cosmetics

## Abstract

Progress in recent years in the field of stimuli-responsive polymers, whose properties change depending on the intensity of a signal, permitted an increase in smart drug delivery systems (SDDS). SDDS have attracted the attention of the scientific community because they can help meet two current challenges of the pharmaceutical industry: targeted drug delivery and personalized medicine. Controlled release of the active ingredient can be achieved through various stimuli, among which are temperature, pH, redox potential or even enzymes. SDDS, hitherto explored mainly in oncology, are now developed in the fields of dermatology and cosmetics. They are mostly hydrogels or nanosystems, and the most-used stimuli are pH and temperature. This review offers an overview of polymer-based SDDS developed to trigger the release of active ingredients intended to treat skin conditions or pathologies. The methods used to attest to stimuli-responsiveness in vitro, ex vivo and in vivo are discussed.

## 1. Introduction

Smart drug delivery systems (SDDS) have been designed to control the release of active molecules into the intended site of action when a biological or a physical stimulus occurs. The interest of such systems can be the control of the release kinetics and/or minimization of side effects [1]. Such systems can be made of stimuli-responsive polymers, the properties of which will change depending on environmental conditions. Depending on the polymer, a very slight modification of the environment can produce a noticeable macroscopic alteration in their characteristics (physical state, shape and solubility, solvent interactions, hydrophilic and lipophilic balances). Light-responsive, temperature-responsive, pH-responsive or redox-responsive polymers can be utilized to prepare such systems [1]. After the specific stimulation, the responses of SDDS vary widely from swelling/contraction to disintegration, favoring the release of encapsulated drugs. The most interesting features of the smart polymers arise from their versatility and sensitivity tuning by means of chemical modifications.

SDDS has been widely explored in oncology, where stimuli are specific to the tumor environment [2,3]. Lately, much effort has been focused on the development of stimuli-responsive systems adapted to the dermatology and cosmetology fields. The delivery of active ingredients (AI) to the skin is a true challenge for researchers trying to combine efficacy with this local and non-traumatic route of administration. SDDS is a promising research path since skin conditions or dermatoses can lead to imbalances in the skin physiological parameters like pH, temperature or redox potential [4,5,6,7]. Moreover, easy access to the skin surface opens a wide field for the use of external triggers including UV, infrared (IR) light and heat [8,9,10]. Smart polymers are then used for two main purposes: the protection of the AI in the formulation and the increase in its efficacy when applied to the skin.

This review sheds a light on the last development in polymer-based SDDS designed for skin application found in the literature. After a reminder of the specificity of drug administration to skin, it will focus on the major stimuli applicable. Finally, it will raise the topic of the assessment of SDDS in triggered release in vitro, ex vivo or in vivo, still in the context of skin application.

## 2. The Skin, A Well-Established Route for Drug Delivery But Still Challenging

Human skin is the largest and the most accessible organ of the human body, making it a promising alternative to oral and injectable drug delivery. Other superior aspects of skin administration are ease of application and enhanced patient compliance, the possibility of local (dermal) and systemic (transdermal) delivery, and avoidance of systemic adverse effects due to drug concentration at a local site [11]. The intention of local delivery is to directly treat cutaneous disorders on the skin surface or within the skin directly beneath the application site, such as eczema or psoriasis. For cosmetic AI, any systemic absorption that may occur is undesirable. Systemic delivery implies application of the drug to the skin to treat systemic disorders.

However, drug delivery by cutaneous route is not a simple task. Human skin is a stratified tissue that can be divided in three functional layers: the epidermis (the outermost layer of the skin), dermis and hypodermis. The outermost structure of the epidermis is the stratum corneum (SC), a region rich in proteins and lipids that minimizes the inlet and outlet of water, oxygen and chemicals. This defensive membrane structure restricts the penetration of active ingredients into the deeper layers of the skin. It is the main component of the so-called skin barrier.

Skin penetration depends on physicochemical properties of the active ingredient (AI) and is favored if the latter meets the following criteria: low molecular weight (<500 Da), satisfactory polarity (log P_o/w_ ≅ 1–3) and low melting point [12,13]. Drug penetration will also vary according to several physio-pathological parameters: skin areas, temperature and skin circulation, skin condition (the existence of lesions increases absorption), and the age of the patient. In view of the diversity of AI and their physicochemical properties, the penetration rules imposed by the composition and structure of the skin limit the cutaneous drug delivery. An active ingredient is never used directly in its pure form; it is integrated into an appropriate dosage form (mainly composed of excipients), which conducts it on the skin and accelerates its diffusion in the epidermis. The rate and extent to which an AI penetrates the skin depend on formulation-related factors [14]. The choice of dosage form will mainly depend on the body area of application of the product, on the nature of the active ingredient of interest, and on the level of penetration needed. Conventional cutaneous drug delivery systems include semisolid and liquid dosage forms. The semisolid dosage forms include ointments, creams, gels, or pastes, while the liquid dosage forms include lotions that may be an emulsion, suspension, or a solution.

A variety of physical or chemical methods has been proposed to overcome skin barrier functions momentarily in order to enhance drug delivery. Physical enhancers employ electrical, thermal (laser ablation), ultrasound, and mechanical effects (microneedles) [15,16]. However, these physical enhancers could severely damage the SC barrier and cause skin drying, irritancy, and hypersensitivity [17,18]. Chemical enhancers are molecules such as surfactants, terpenes, propylene glycol, fatty acids, alcohols, sulfoxides, etc. [19,20]. Many potent enhancers that disrupt the skin barrier also disrupt viable cells and cause toxicity. Moreover, enhancer effects are hard to predict (they are drug-specific and differ on animal and human skin) [20].

To facilitate drug delivery by topical application, smart drug delivery systems (SDDS) can be used as an alternative or complement to the above-mentioned skin delivery technologies.

## 3. Polymer-Based Smart Drug Delivery Systems for Topical Applications

### 3.1. Types of SDDS Formulations for Topical Applications

Polymer-based nanogels or hydrogels are the most widely described systems when it comes to developing smart systems for skin applications.

**Figure 1:**
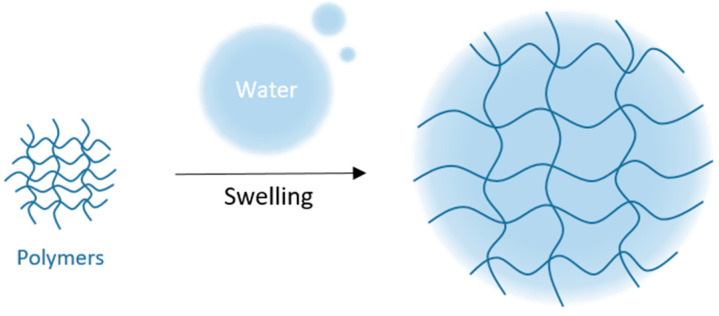
Hydrophilic gel formation by swelling of polymers in water.

Hydrophilic gels, also called hydrogels, are common pharmaceutical forms used to deliver drugs to the skin. Gels are transparent or translucent semisolid formulations containing a high ratio of solvent/gelling agent. When dispersed in an appropriate solvent, gelling agents merge or entangle to form a three-dimensional colloidal network structure, which limits fluid flow by entrapment and immobilization of the solvent molecules. Hydrogels have been used for a long time in dermatology and cosmetology fields due to their biocompatibility, their good spreading and adherence to the site of application, their long resident time on the site of action facilitating diffusion of the AI in the skin. The core of a hydrogel is a polymeric channel system, which may be formed through physical or chemical cross-linking of homopolymers or copolymers [21], resulting in swelling when subjected to an aqueous surrounding (Figure 1).

Thanks to their small size (1–100 nm) and physicochemical composition, polymeric nanosystems offer many advantages over free drug solution: (1) they protect the drug from premature degradation, (2) they prevent drug from prematurely interacting with the biological environment, (3) they enhance absorption of the drug into a selected tissue such as skin, (4) they control the pharmacokinetics and drug tissue distribution profile, (5) they improve intracellular penetration, (6) they solve formulation problems [22,23]. Polymeric nanosystems include nanomicelles, polymersomes, nanoparticles, and nanogels [24]. Nanogels are three-dimensional hydrogel materials in the nanoscale size range [25].

**Figure 2:**
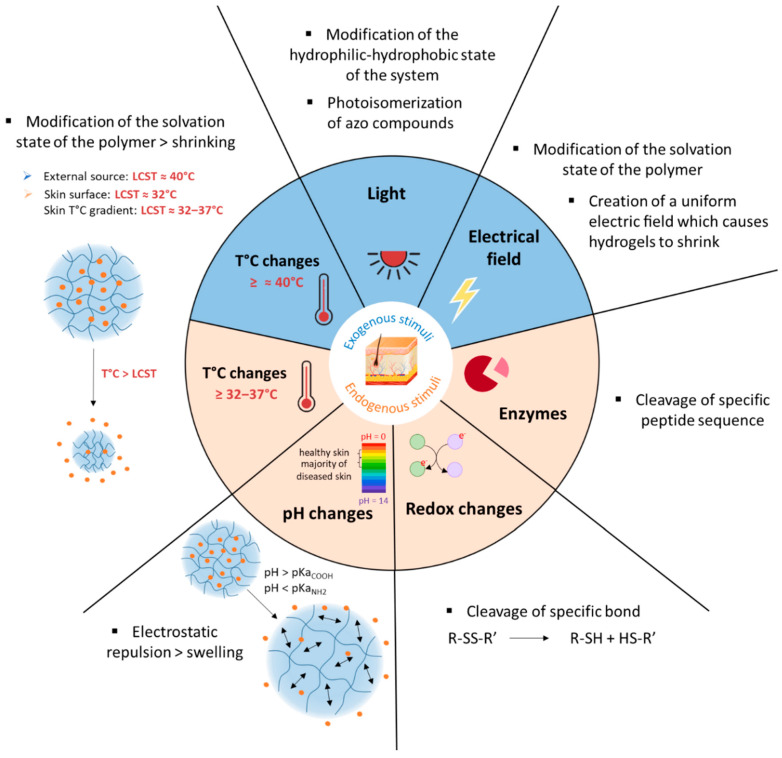
Schematic overview of stimuli and their mode of action, applied to the design of smart drug delivery systems for skin applications.

The preparation of these smart gels is obtained by using stimuli-responsive polymers. The associated stimulus will cause a conformational or structural change in the gels, which is mediated by various factors including, but not limited to, transition in the temperature below or above its lower critical solution temperature (LCST) or the ionization of acidic or basic functional groups on the polymer chains. These changes also alter the hydrophilicity and/or hydrophobicity of the gels or, in other words, the extent of interaction of the system with water molecules. It is mostly manifested in the form of swelling, shrinking or disintegration of the gel network, which in turn causes responses like release of the entrapped cargo. Figure 2 compiles the different stimuli and their mode of action used in the skin area.

In a general way, SDDS can be constructed with polymers of synthetic, semi-synthetic or natural origin. Although a number of synthetic biodegradable polymers have been developed for biomedical applications, the use of natural biodegradable polymers remains attractive because of their abundance in nature, good biocompatibility and ability to be readily modified by simple chemistry.

#### 3.2. pH-Responsive Delivery Systems

##### 3.2.1. Skin pH and Its Variations

Maintenance of a low skin pH (4–6) is vital for many normal human skin functions (homeostasis, integrity/cohesion of the SC, and desquamation) [18,26]. Deviations in skin pH, notably its elevation, lead to disturbances of the skin barrier and the skin microbiome and consequent infection and inflammation. Examples are atopic dermatitis (AD) [6,27,28,29], irritant contact dermatitis [30], candidiasis [31] and acne [32]. pH variation is also observed in the wound healing process, which can be divided into four stages: hemostasis, inflammation, proliferation, and remodeling. The shift from the inflammatory stage to the proliferative stage results in a gradual pH increase from 5.7 to 7.6 [33]. As several skin diseases are associated with an imbalance in the pH of the skin, systems based on pH-responsive polymers have been explored for controlled drug release in the area to be treated.

##### 3.2.2. Mechanisms of pH-Responsiveness in Smart Polymeric Systems

All pH-responsive polymers are based on functional groups that can either accept or release a proton in response to changes in the pH of the environment. pH-responsive polymers are then classified into two types: anionic and cationic polymers (Table 1).

**Figure 3:**
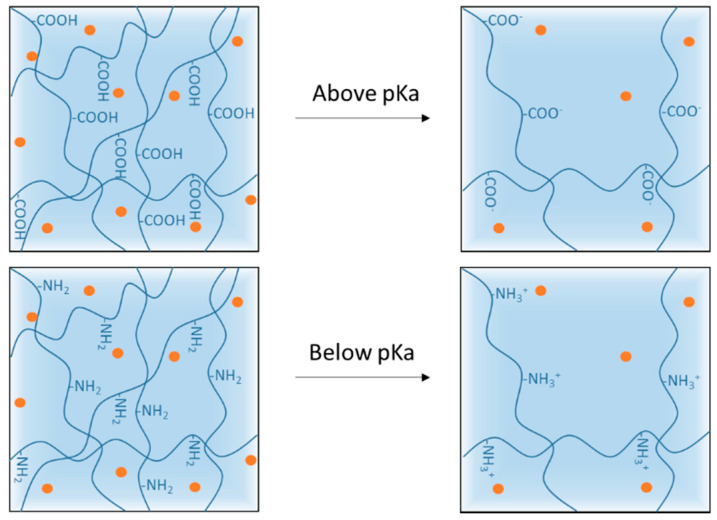
Schematic illustration of pH-responsive hydrogel response to surrounding medium pH variations (above: anionic polymer, below: cationic polymer).

Anionic polymers are often composed of carboxylates i.e., of carboxylic acid groups ionized at pH above their pKa ≈ 4. Cationic polymers are composed of amine groups ionized to NH_3_^+^ form at pH below their pKa ≈ 6.5. The ionization of the functional groups presented along the backbone and side chains of the polymer leads to a conformational change in the polymer resulting in its swelling/shrinking or dissolution [42,62,63]. For hydrogels and nanogels, rapid ionization leads to rapid swelling of the system due to electrostatic repulsion inside the polymer network. This swelling increases the size of the pores in the system, which causes or accelerates the release of the cargo on the target site (Figure 3).

##### 3.2.3. pH-Responsive Systems Based on Anionic Polymers

As several skin diseases are associated with a rise in pH (e.g., wounds, dermatitis) compared with healthy skin, anionic polymer systems are more relevant and explored for smart topical treatment of skin diseases. For example, acrylic acid, methacrylic acid, maleic acid, itaconic acid, cellulose derivatives, hyaluronic acid, etc., have already been explored to develop pH-responsive systems for dermal applications.

**Poly(acrylic acid).** Poly(acrylic acid) (PAA), also known as carbomer, is of particular interest because it is biocompatible and biodegradable. PAA is a synthetic high-molecular weight polymer of acrylic acid with a pKa of ~4 [64]. Acrylic acid-based polymers have been used to develop SDDS sensitive to the rise in pH of wounds. For example, Koehler and co-workers developed pH-regulated hydrogel dressings based on alginate, poly(ethylene glycol) diacrylate and acrylic acid (AA). Important properties of wound dressings, such as swelling capacity and mechanical strength, were adjusted by varying the AA concentration. Swelling behavior could help to absorb exudates and provide mechanical resiliency to the delivery system at the biological site of action. The most promising formulation (hydrogels with 0.25% acrylic acid) was tested on injured human skin constructs and increased keratinocyte growth in the wound by 164% [54]. Zhu and co-workers reported the preparation of pH-responsive hydrogels for wound dressing application, comprised of peptide-based bis-acrylate and acrylic acid. The authors reported that the hydrogels possessed a tough and non-toxic nature with pH-dependent swelling behavior efficiently releasing the antibacterial drug triclosan when the preparation became alkaline [55].

**Poly(methacrylic acid).** Poly(methacrylic acid) has a pKa of ~4.5 [64]. Methacrylic-acid-based polymers have been used to develop systems sensitive to the rise in pH of several skin diseases. Eudragit^®^ L100 (methacrylic acid-*co*-methyl methacrylate, 1:1) is a biocompatible polymer known to be soluble above pH = 6, a pH close to the skin pH in many inflammatory skin diseases, which makes it a good candidate for developing pH-responsive particles [49,56,63,65]. For example, Sahle et al. used Eudragit^®^ L100 to develop pH-responsive nanoparticles (NPs) that would release dexamethasone in a controlled manner on the skin but dissolve in hair follicles [49,56]. Rizi et al. developed hydrocortisone-loaded Eudragit^®^ L100 microparticles that deliver essentially no drug at normal skin pH. The delivery can be triggered and targeted to atopic dermatitis skin where the pH is increased [65]. Eudragit^®^ L100 was also used by Dong P. et al. to develop dexamethasone-loaded nanoparticles. Results showed that these NPs improved cutaneous penetration and controlled the release of a lipophilic drug, especially on barrier-disrupted skin [63]. Eudragit^®^ S100 (methacrylic acid-*co*-methyl methacrylate, 1:2) and Eudragit^®^ L100-55 (methacrylic acid-co-ethyl acrylate, 1:1) were also explored to develop dexamethasone-loaded particles [49].

**Poly(itaconic acid).** Itaconic acid (IA) is produced industrially by the fermentation of carbohydrates such as glucose or molasses. IA is a weak acid with two carboxylic groups and two pKa constants, pKa_1_ = 3.85 and pKa_2_ = 5.45, making it an attractive polymer for the development of SDDS. For example, Vuković and co-workers designed biocompatible pH-responsive hydrogels based on 2-hydroxyethylacrylate and itaconic acid (IA) for the treatment of skin/wound infections. The swelling of the hydrogels is significantly dependent on the content of hydrophilic IA [62].

**Cellulose derivatives.** Cellulose is the most abundant polysaccharide found in nature. Unsurprisingly, it is the main raw material for many semisynthetic cellulose derivatives. To add a pH-responsive character to cellulose, carboxyl groups (-COOH) were incorporated into the structure of the polymer by simple chemistry. For example, phthalate derivatives of cellulose were produced and contain a carboxyl group with a pKa of approximately 4.3. Sahle and co-workers used hydroxypropyl methyl cellulose phthalate (HPMCP) and cellulose acetate phthalate (CAP) to develop pH-responsive dexamethasone-loaded nanoparticles [49]. Another cellulose derivative is carboxymethyl cellulose (CMC), formed when cellulose reacts with mono chloroacetic acid. CMC contains carboxyl groups with a pKa of approximately 4.3. CMC is widely used owing to its high biodegradability, non-toxicity, and biocompatible properties. Park and co-workers prepared a pH-responsive hydrogel based on a CMC and 2-hydroxyethyl acrylate (HEA) as a grafting agent [48]. This hydrogel was investigated as a transdermal delivery system for naringenin, a drug for treatment of atopic dermatitis (AD). The swelling ratio of the hydrogel increased as the grafting and the crosslinking density decreased, and it also increased at pH 7.5 and 8.5 (compared with pH 5.5). The hydrogel hydrates the skin, which temporarily interferes with the barrier function of the skin and allows the drug to penetrate the skin. Therefore, this novel pH-responsive hydrogel has potential applications in the treatment of various skin lesions caused by pH imbalance, such as AD.

**Chitosan derivatives.** Chitin is the most abundant polysaccharide in nature after cellulose. The partial or full deacetylation of *N*-acetyl-glucosamine moieties of chitin leads to the formation of chitosan, also considered a natural, nontoxic, biodegradable and bio-compatible polymer [66]. Chitosan has natural pH-responsive properties due to the protonation-deprotonation balance of amino groups. After a carboxymethylation reaction of chitosan, carboxymethyl chitosan (CmCHT) is produced, and the carboxyl groups added have a pKa of around 4.5. The primary interest of this chemical modification was to increase the water solubility of chitosan in alkaline pH. However, in view of the interest that a carboxyl group can represent in the development of a system sensitive to the rise in pH, this cellulose derivative has been used to develop SDDS. For example, Jeong et al. synthesized pH-responsive hydrogels by grafting 2-hydroxyethyl acrylate (HEA) onto CmCHT [44]. This hydrogel CmCHT-g-pHEA was investigated as a transdermal delivery system for nobiletin, which is effective in fighting acne. Since the amine group of the chitosan derivative is involved in the structure of the hydrogel network, only the carboxyl group will be at the origin of the pH-responsive behavior. The swelling ratio of the hydrogel increased at high pH, and in vitro skin permeation experiments attested that the CmCHT-g-pHEA hydrogel improved the transdermal delivery of nobiletin. In conclusion, the newly synthesized CmCHT-g-pHEA hydrogel has potential as a non-toxic transdermal delivery carrier for treatment of skin lesions such as acne.

**Hyaluronic acid.** Hyaluronic acid (HA) is a natural and endogenous polysaccharide that plays important physiological and biological roles in the human body. Nowadays, HA is emerging as an appealing starting material for hydrogel design due to its biocompatibility, native biofunctionality, biodegradability, non-immunogenicity, and versatility. Given that HA contains carboxyl groups with a pKa in the range 3–4, it has been used to develop pH-responsive systems. For example, Kwon and co-workers investigated a pH-responsive hydrogel based on hyaluronic acid to deliver isoliquiritigenin (ILTG), an antimicrobial therapeutic agent, for acne growth inhibition [45]. Due to the significantly increased swelling of the hydrogel carrier, the ILTG could be released substantially at around pH = 7, where colony formation of acne is most active. The hydrogel was found to exhibit excellent permeability of ILTG into the skin, which penetrated mostly via the follicular pathway.

**Alginate.** Alginate is a natural polysaccharide, a copolymer of β-L-guluronic and α-D-mannuronic acid blocks, each with a carboxyl group (pKa of 3.65 and 3.38, respectively). Since alginate is biocompatible, non-toxic and non-immunogenic, it constitutes a good component for the production of a SDDS. For example, Shi and co-workers develop alginate-based microparticles that showed tunable compositions and were pH-sensitive for sustainable release of drugs in wound healing applications [51].

**Agarose derivatives.** Agarose is a natural polysaccharide extracted from red algae with repeating units of 1,3-linked-D-galactose and 1,4-linked 3,6-anhydro-1-galactose residues. Due to its good biocompatibility, agarose have seen employed widely in cosmetic and biomedical applications. To confer pH-responsive properties to agarose, it can be chemically modified through the precise oxidation of the primary alcohol of the D-galactose into carboxylic acid. This chemical modification provides a novel class of materials named carboxylated agarose (CA). In work done by Ninan et al., novel CA/tannic acid hydrogel scaffolds cross-linked with zinc ions were designed for the pH-controlled release of tannic acid for wound dressings [50].

**Keratin.** Keratin are proteins derived from human hair, wool, feathers, horns, hooves and nails. Due to their unique characteristics of bioactivity, biocompatibility, biodegradability, and natural abundance, keratin have been widely developed in wound healing applications [67]. Carboxyl groups are present in keratin structure, and the mechanism of swelling in response to the media pH change would be a protein structural reorganization driven by carboxyl protonation/deprotonation [68]. Villanueva et al. developed a smart antibacterial biomaterial based on a keratin hydrogel with pH-dependent behavior and Zinc Oxide (ZnO) nanoplates as a biocide agent. Keratin hydrogels swell at basic pH, such as a bacterial contaminated media, leading to the release of ZnO nanoparticles. The results are encouraging for wound dressing applications [52].

##### 3.2.4. pH-Responsive Systems Based on Cationic Polymers

One strategy of designing SDDS based on cationic polymers (containing amine groups) is to prevent the initial burst release of active ingredients into the dosage form at neutral pH, and once in contact with the more acidic skin (<pKa of amine groups), the drug release is triggered [40,43]. Another strategy is based on the pH imbalance between the tumor microenvironment and normal skin. The accumulation of lactic acid in the tumor microenvironment leads to a decrease in cell pH from 7.5 to 4.5–6.5 [69]. This pH imbalance is already utilized to trigger drug release from pH-responsive cationic systems in response to the acidic environment within the cancer cell [69,70,71]. For example, Sabitha et al. and Sahu et al. designed pH-responsive chitosan-based nanogels for topical therapy of skin cancers [34,35,36,37,38,39]. However, for topical application, this strategy is complicated to implement given the already acidic pH of healthy skin.

In a pH-responsive system, drug release is often associated with its stimulated swelling. However, in one pH-responsive system study, the release of the drug was associated with a shrinking of a cationic system. For example, Zhu et al. studied pH-sensitive methacrylated chitosan (MAC) hydrogels, and significant decreases in the swelling ratios were observed when the hydrogel was exposed to increasingly alkaline environments (pH 3, 5, 7.4 and 9). Indeed, the amino groups of MAC (pKa = 6.5) will be deprotonated when the environmental pH is higher than the functional group’s pKa. Therefore, the number of positive charges in the network of chitosan-based hydrogel decreases as the pH increases, which results in the shrinking of the system (because there are less electrostatic repulsions). Results showed that MAC hydrogels have a potential application in responding to specific wound healing stages by pH dependence and accelerate wound healing by the liberation of antibiotics and anti-inflammatory drugs [42].

#### 3.3. Thermoresponsive Delivery Systems

##### 3.3.1. Application Strategies of a Thermoresponsive System for Skin Delivery

Temperature-induced drug release is among the most investigated strategies in biomedicine, given the wide range of applications where temperature variations are naturally present. For cutaneous delivery, several strategies are available:

- Using the temperature difference between the formulation (usually at room temperature) and the skin surface (32 °C). For example, when thermoresponsive system is applied to the skin surface and reaches its temperature, it will quickly release the drug [72,73].
- Using the thermal gradient of the skin (32–37 °C) to deliver drugs. This strategy is usually adapted to nanosystems. The surface temperature (32 °C) prevents immediate drug release, and it is only when nanovectors finally reach deeper layers of the SC (37 °C) that the drug is released [58,74,75,76].
- Using the temperature imbalance between healthy skin and injured skin to trigger the release of active ingredients, specifically on the injured site concerned, by application of the active ingredient [77]. For example, it was shown that chronically infected wounds show a temperature 3 °C to 4 °C higher than normal skin [4].
- Elevating the temperature of the region to be treated artificially using an external thermal trigger, e.g., heating patch or infrared lamp [10].

The temperature stimulus is also used to form gels in situ [78]. Typically, aqueous solutions of hydrogels used in biomedical applications are liquid at ambient temperature and gel at physiological temperature (37°C). Herein, this sol-gel transition is used in particular for wound covering, since solutions can be applied easily using a syringe and then directly form a firm overlay covering the wound upon contact with the skin [79,80,81]. In this case, the stimulus is not used to trigger the release but to facilitate the use of the medicine.

##### 3.3.2. Mechanisms of the Temperature Responsiveness of Smart Polymeric Systems

**Figure 4:**
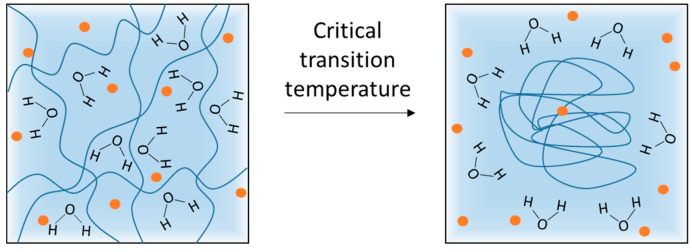
Schematic illustration of LCST hydrogel behavior.

Thermoresponsive polymers exhibit a volume phase transition at a certain temperature (VPTT), which causes a sudden change in the solvation state, conformational state and, consequently, water-solubility [82]. Polymers that become insoluble upon heating have a so-called low critical solution temperature (LCST). Polymers that become soluble upon heating have an upper critical solution temperature (UCST). For topical applications, LCST polymers have been extensively studied because their volume phase transition can occur between room temperature (25 °C) and body temperature (32–37 °C). This means that contact of the systems with the skin/body temperature exceeding their LCST causes a rapid decrease in the volume of the system, resulting in a fast expulsion of fluids in the matrix that could be accompanied by the release of loaded hydrophilic or hydrophobic active ingredients [83]. Thermoresponsive hydrogel can be made by cross-linking polymers that display LCST behavior (Figure 4). Below the LCST, the polymers are water soluble, whereas above the LCST, they become increasingly hydrophobic and insoluble, leading to gel formation. This temperature-dependent sol-gel transition can be experimentally verified by a number of techniques such as spectroscopy, differential scanning calorimetry, and rheology [84,85,86] and will depend on the chemical nature and polymer concentration. It is then possible to chemically tune the LCST of thermoresponsive polymers according to the requirements of the applications under investigation [87].

##### 3.3.3. Thermoresponsive SDDS for Cutaneous Administration

Various thermoresponsive polymers are used to develop thermoresponsive systems, usually hydrogels or nanogels, for the controlled drug delivery by cutaneous route (Table 2).

**Poly(*N*-isopropylacrylamide) (PNIPAM).** Over the past few years, PNIPAM has appeared in the literature with increasing frequency. PNIPAM-based hydrogels, when heated above the LCST (≈32 °C), undergo a reversible phase transition from a swollen hydrated state to a shrunken dehydrated state, losing their volume and releasing their content. One of the first examples was the development of a PNIPAM drug-loaded hydrogel wound dressing with thermoresponsive, adhesive, and absorptive functions [99].

PNIPAM may be co-polymerised with other monomers, such as butyl acrylate (BuA) [100], acrylamide [87], or ethylene glycol [101]. Copolymerization of NIPAM with different monomers thus allows tuning of the properties, especially LCST.

PNIPAM may be polymerized from dendritic polyglycerol (dPG). dPG is a hydrophilic macromolecule that acts as a macro-crosslinker and allows the growth of multiple thermoresponsive polymer chains such as PNIPAM. dPG allows easy preparation of a monodisperse and stable nanostructure with good aqueous solubility and high biocompatibility [107,108]. For example, Witting et al. designed 200 nm dPG nanogels grafted with PNIPAM for protein encapsulation [76]. These NGs exhibited a thermal trigger point at 35 °C, which is favorable for cutaneous applications. Indeed, at ≥35 °C, the particle size was instantly reduced by 20%, and 93% of the protein was released. These thermoresponsive nanogels (tNGs) are promising topical delivery systems for biomacromolecules. Osorio et al. synthesized nanocapsules (NCs) using modified silica nanoparticles as sacrificial templates and dPG as a macrocrosslinker, combined with different ratios of PNIPAM and PNIPMAM. The VPTT of these NCs is close to 40 °C and allows for drug delivery at elevated temperatures triggered by an external heat source [95].

Ugazio and coworkers developed mesoporous silica nanoparticles (MSNs) grafted with PNIPAM and loaded with quercetin. This system can be considered as a promising approach to controlling the skin delivery of antioxidants using a thermal trigger [97].

PNIPAM polymers are nontoxic and biocompatible, but NIPAM monomers may have some toxic effects [109].

**Poly(ethylene glycol) methacrylate.** Thermoresponsive methacrylate and acrylate polymers with short oligo(ethylene glycol) side chains have been found to possess properties that are comparable with PNIPAM. In particular, Lutz et al. have reported an interesting thermoresponsive polymer, poly(di(ethylene glycol) ethyl ether methacrylate) (PDEGMA), with a LCST close to 28 °C [110,111]. Importantly, PDEGMA and its copolymers are low-toxicity, antifouling, and as such have been successfully used to replace PNIPAM in a number of biomedical applications. Asadian-Birjand et al. have developed tNGs for inflamed skin treatment. tNGs were synthesized through the free radical polymerization of acrylated dendritic polyglycerol (dPG-Ac), ethylene glycol methacrylates di(ethylene glycol)methyl ether methacrylate (DEGMA) and oligo ethylene glycolmethacrylate (OEGMA). The volume phase transition temperature (VPTT) is around 36–40 °C, slightly higher than the temperature of healthy skin. It is expected that once the tNGs surpass their VPTT, for instance in inflamed skin areas, their polarity will change from a hydrophilic to a hydrophobic state, improving the interaction with the hydrophobic structures of the stratum corneum that would result in a better penetration in the skin [77].

**Polyglycerol derivatives.** In the case of linear polyglycerol (PG), a lower critical solution temperature (LCST) can be induced by hydrophobic modification of the hydroxyl groups. The hydroxyl moieties can be converted into various functional groups like ethers, esters, urethanes or carbonates [112]. For example, thermoresponsive and highly biocompatible poly(glycidyl ether)s copolymers composed of ethyl and methyl glycidyl ether were synthesized. In Giulbudagian’s studies, click chemistry was used to graft these poly(glycidyl ether)s copolymers on dendritic polyglycerol (dPG) and develop thermoresponsive nanogels (tNGs_tPG) for topical delivery. The cloud point temperature (Tcp) of these tNGs_tPG occurs at 32 °C. While tNGs are swollen with water below their Tcp, the thermoresponsive polymers of the tNGs undergo a reversible transition in water solubility when exposed to greater temperatures, resulting in gel shrinking and the expulsion of water and drugs [74,113]. Specially, it was shown that polyglycerol-based thermoresponsive nanogels cause high local hydration in the stratum corneum (SC), altering the organization of both lipids and proteins, thus increasing the skin penetration of the released cargo [114]. Rancan et al. also investigated a tNG based on dendritic polyglycerol (dPG) and poly(glycidyl ether)s copolymers with a Tcp of 34 °C. They successfully demonstrated the penetration of tNGs in the SC of both intact and disrupted skin models. The studies showed enhanced penetration of tNGs with a release of dye in the epidermis on a thermal trigger by infrared radiation. Additionally, in barrier-disrupted skin, considerable quantities of the tNGs and dye were detected in both epidermis and dermis and thus showed promising applications for the treatment of inflammatory skin diseases [10].

**Poly(*N*-vinylcaprolactam) (PVCL).** PVCL is a temperature-responsive polymer with an LCST in the physiological range (~32-37 °C). PVCL polymers attract high interest because of PVCL solubility in aqueous and organic solutions, as well as strong hydrophilicity and nontoxicity compared with PNIPAM [115,116]. Zavgorodnya and coworkers prepared temperature-responsive hydrogel films of PVCL for topical drug delivery. PVCL-based hydrogel loaded with sodium diclofenac, an anti-inflammatory drug used for osteoarthritis pain management, provides sustained permeation of this drug through an artificial skin membrane. The cumulative amount of diclofenac transported at 32 °C from the PVCL-based hydrogel was higher than that from the PVCL-based hydrogel at 22 °C, validating the interest of such a system for cutaneous drug delivery [104].

**Oligo(-ethylene glycol)-decorated polyisocyanopeptide (PIC).** The gelation temperature of PIC is 20 °C. At temperatures below 20 °C, the polymer solution is a free-flowing liquid, whereas above 20 °C the viscosity dramatically and rapidly increases whereby the polymer solution turns into a hydrogel. The PIC-based gel mimics the fibrous structure and the mechanical properties of natural extracellular matrix materials. These biomimetic PIC hydrogels were used to develop wound dressings. Indeed, PIC solutions gel upon contact with body heat and stay adherent to the wound without additional support [102].

**Poloxamers.** Triblock copolymers poly(ethylene oxide)-*b*-poly(propylene oxide)-*b*-poly(ethylene oxide) (PEO-PPO-PEO), also known as Poloxamers or Pluronics^®^, are an important group of synthetic polymers with a thermoreversible behavior in aqueous solutions. Poloxamers are approved by Food and Drug Administration for use in humans and have been extensively used in several biomedical applications because of their biocompatibility. Pluronic^®^ F127 (PF127), the most commonly used, is composed of PEO units (70%) and PPO units (30%) and transformed from a low-viscosity solution to a semisolid gel upon heating to body temperature at concentrations of >20% *w*/*v* [117]. In the dermatological field, the sol-gel transition of PF127-based hydrogels is commonly used for wound healing [79,80,81,91,92]. For example, Heilmann et al. developed a thermosensitive morphine-loaded PF127 hydrogel that provides a moist environment to the wound, which is known to facilitate the healing process. Indeed, at temperatures of about 4–8 °C, the formulation can be easily handled due to its liquid aspect. However, once in contact with the skin surface (32 °C), the formulation undergoes a sol-to-gel transition [80]. Poloxamers can be modified by chemical grafting to modulate the gelation temperature [93,94]. Pluronic^®^ F127 can be combined with carboxymethyl cellulose (CMC) to increase the porosity of the matrix to facilitate the diffusion of drugs out of it [89,90].

#### 3.4. Other Stimuli-Responsive Delivery Systems

##### 3.4.1. Redox-Responsive Systems

Lately, a lot of efforts have been focused on the development of redox-responsive systems [118,119,120]. It is well established that the pathological consequences of inflammation result in the formation of reactive oxygen species (ROS) and other oxidants, causing an oxidative imbalance (stress). ROS often induce redox adaptation in response to the continued oxidative stress, leading to an up regulation of glutathione (GSH) and other antioxidant molecules. Since disulfide bonds are sensitive to GSH, they were incorporated into nanocarriers for smart drug delivery within the cancer cell, where high concentrations of GSH were reported [121,122,123].

The strategy of disulfide bonds incorporation was used by our group [124] in order to develop redox-responsive nanoparticles for topical applications. Briefly, a mixture of poly(lactide) (PLA) and redox-responsive poly(ethylene glycol)-block-poly(lactide) (PEG-block-PLA) containing a disulfide bond was synthesized in three steps. Retinol, an anti-aging agent very common in cosmetics, was loaded into these smart nanocarriers. Results showed that increased GSH activity could favor the nanocarrier’s cleavage and thus enhance the release of hydrophobic drugs.

##### 3.4.2. Enzyme-Cleavable Systems

The skin is an organ with high enzyme activity (e.g., Cytochrome P450), which is important to consider when designing dermally administered drugs. Indeed, SDDS could control the delivery of active ingredients based on the biological environment, including the enzymatic environment. For example, during the wound-healing cascade, matrix metalloproteinases (MMPs) are secreted by the cellular components. Kim et al. described polymer fiber patches, from which recombinant EGF was engineered, as releasing only in the presence of wound-healing cues, e.g., MMP-9. Results showed that the EGF-loaded responsive fiber significantly promoted proliferation and migration of human keratinocytes in the presence of the biological trigger MMP-9 [125].

##### 3.4.3. Electro-Sensitive Systems

An electrical field in the form of an external stimulus allows for precise control over the magnitude of the current, the duration of electrical pulses and the interval between pulses. Delivery systems exploiting this external stimulus are prepared from polyelectrolytes, which are polymers that contain a relatively high concentration of ionizable groups along the backbone chain. Under the influence of an electric field, electro-responsive hydrogels generally shrink or swell, and this property has allowed for their application in drug delivery systems [126]. Im et al. were some of the first to develop an electro-sensitive transdermal drug delivery system. They developed an electro-sensitive system by the electrospinning of polyethylene oxide/pentaerythritol triacrylate/multi-walled carbon nanotubes, and they observed a correlation between drug release and applied voltage [127].

Oktay et al. designed an electro-sensitive hydrogel based on poly(3,4-ethylenedioxythiophene) (PEDOT). They demonstrated that the model drug was released from the polymeric matrix as a result of the hydrogel shrinking upon exposure to an electric field. This formulation could be used as a drug carrier for electric-stimuli controlled delivery in the treatment of skin cancer [128].

#### 3.5. Dual Stimuli-Responsive Systems

In addition to the single response polymer, it is also possible to design and engineer materials that respond simultaneously to a combination of stimuli (e.g., temperature, pH, redox potential). By combining two properties, this creates a polymer that is more specific and controllable.

The development of systems responding simultaneously to temperature and pH changes is a wide and interesting area of research for the development of specific drug carriers. For example, thermo-sensitive PNIPAM may be combined with pH-responsive monomers, such as methacrylic acid (MAA), acrylic acid (AAc) or hyaluronic acid (HA) [46,53,59,96]. For example, Banerjee and co-workers developed a poly(NIPAM-co-acrylic acid) hydrogel that can be used for the sustained release of growth factors for a better healing response [53]. Kim et al. developed PNIPAM/HA hydrogel that efficiently delivers luteolin to the epidermis and dermis, for skin relief in psoriasis [46].

Indulekha et al. designed a thermoresponsive gel as an on-demand transdermal drug delivery system for pain management. They grafted pH-responsive chitosan onto thermoresponsive poly(N-vinyl caprolactam) (PVCL). Drug release from the gel and drug permeation through the skin were better at 39 °C and for a pH of 5.5. Patients can administer themselves with a pulse of drugs through the application of a heat pad over the transdermal drug delivery system, whenever pain is experienced [103]. This could be an alternative to implantable pumps for pain management.

Yamazaki et al. studied the effect of dual-responsive liposomes on melanocytes via the transdermal route. Methoxy diethyleneglycol methacrylate (MD) and methacrylic acid (MAA) were used, respectively, for temperature-sensitivity and pH-sensitivity. The content release from copolymer-modified liposomes was enhanced under acidic pH and body-temperature conditions (35–37 °C), corresponding to a skin environment. Owing to the deep penetration, the modified liposomes delivered antioxidants or UV-protective agents to melanocytes residing in deep skin tissues [58].

Jung et al. used the pH and temperature imbalance between the formulation and the lesions of atopic dermatitis to trigger active ingredient release from cationic nanocarriers [40]. In this study, ceramide-imbedded PLGA nanoparticles were developed with chitosan coating (Chi-PLGA/Cer). The chitosan coating was formed by electrostatic interactions with PLGA and prevented the initial burst release of ceramide. At low pH, the positive-rich condition of chitosan weakened the electrostatic interactions, leading to its removal from PLGA/Cer nanoparticles and resulting in a controlled release of ceramide with additional driving force from the coiled structure of PLGA at around 36.5 °C. They successfully treated a sodium dodecyl sulfate (SDS)-induced model of atopic dermatitis in rats.

Recently, Soriano-Ruiz and co-workers designed a Pluronic/Chitosan/Hyaluronic-based vehicle, including three biological antioxidant molecules, aimed at improving the treatment of skin burns [47].

The development of redox/pH-responsive systems is widely studied because they are triggered by GSH increase and low pH, which are typically significant in inflamed/tumor tissues. However, these studies are rare when they deal with transdermal therapy. Mavuso et al. synthesized a dual pH/redox responsive copper-glyglycine-prednisolone succinate loaded nanoliposomal sludge for transdermal drug delivery [57]. In vitro, ex vivo and in vivo results confirmed the unique pH/redox responsive properties of the system. Nanoliposomal sludge has significant potential for application in chronic inflammatory conditions such as tumor necrosis factor-receptor associated periodic syndrome.

## 4. Proof of Concept: Demonstration of the Stimuli-Responsiveness of the SDDS

Vehicles developed for topical application must respect the following rules: be non-toxic, not irritating to the skin, and have good biocompatibility. Regarding SDDS strategy, several additional investigations are required to prove the stimulus-responsiveness of the system and to check that this sensitivity plays a role in the release of the active ingredient and its penetration in the skin. Indeed, the fact that a system is made of or contains a stimuli-responsive polymer does not mean that it will itself become responsive to the same stimulus or to the same intensity of the stimulus.

### 4.1. Physico-Chemical Characterization of the Stimuli-Responsiveness

#### 4.1.1. Characterization Methods Specific to Thermoresponsive SDDS

Thermoresponsive gels are polymer solutions that transition to a gel state upon an increase in temperature above a critical point. In the articles reviewed here, such sharp changes in properties were followed by related techniques, i.e., viscometry [94], turbidimetry [53], light scattering [77], and calorimetry [100], to identify the phase transition temperature. Some of these techniques are adapted to the characterization of polymer solutions or hydrogels.

**Tcp determination by turbidimetry.** This technique is most widely used to determine the cloud point temperature (Tcp) of thermoresponsive polymer solutions. Tcp refers to the temperature at which the phase transition of a polymer solution at a specific concentration occurs from the soluble state to the collapsed aggregated state, accompanied by clouding of the solution. Briefly, a solution of the polymer is prepared in water, filled into a suitable cuvette and placed in the spectrometer. A temperature program is applied to heat the solution, and the transmittance of light through the solution is constantly measured. Banerjee and co-workers used this method to determine the Tcp value of poly(NIPAM-co-AAc) solution, calculated from the normalized transmittance vs. temperature curve at 50% transmittance. A rapid decrease in the transmittance was observed between 33–37 °C [53].

**Particle size distribution analysis by Dynamic Light Scattering (DLS).** DLS is a non-invasive, well-established technique for measuring the size and size distribution of molecules and particles from below 5 nm to several microns. DLS measurements of the polymer solutions can be performed at different temperatures to follow the volume phase transition temperature (VPTT). Below the VPTT, polymer chains exist as individually dissolved polymer chains with a small hydrodynamic radius, and above the VPTT, polymer chains are partially dehydrated, leading to collapse and agglomeration to form particles of a larger size. Compared with other techniques, DLS provides direct information on the particle size of the polymers, allowing for accurate determination of the onset of the phase separation by the appearance of polymer agglomerates, even when they do not yet cause clouding of the solution. For example, DLS was used to determine the VPTT of thermoresponsive nanogels. At 30 °C, the size is close to 50 nm, but at 36 °C, the size increased to 350 nm, which attests to the thermosensitivity of the system up to a temperature of 36 °C, slightly higher than the temperature of healthy skin [77].

**Phase transition temperature measurement by Differential Scanning Calorimetry (DSC).** DSC is a technique used to investigate the response of polymers to heating. The sudden change from hydrophilic to hydrophobic behavior of the same polymer is based on the loss of the specific hydrogen bonds between the polymer and the surrounding water molecules. This change in hydrophilic-hydrophobic balance of a thermoresponsive polymer is an endothermic process that could be examined by calorimetric methods. Lopez and co-workers used the DSC method to determine the phase transition temperature of PNIPAM-based microgels and observed an endothermic transition at 34 °C [59].

#### 4.1.2. Swelling/Shrinking Studies

Regarding the majority of the smart systems described previously, their sensitivity to stimulus leads to a swelling/shrinking of the system, leading to the release of the AI. Swelling/shrinking analysis could be performed using the DLS method. Indeed, it is possible to follow the hydrodynamic diameter/particle volume as the function of stimulus application (temperature or pH range) [49,56,59,73,77,99,100]. Since DLS analysis must be performed on transparent liquids, it is not available for concentrated or turbid systems such as opaque hydrogels.

For hydrogels, the swelling behavior could also be studied by comparing the weight of the dry vs. wet pellet/hydrogel [35,42,44,45,54]. Briefly, dried hydrogels are completely immersed in swelling medium, then weighed after the excessive solution on the surface is blotted. Thanks to this method, Kwon and co-workers showed that the swelling ratios of anionic polymer-based hydrogels vary from more than 1000% in acidic conditions to more than 3000% in alkali conditions [45].

### 4.2. In Vitro Triggered Drug Release Studies

The aim of smart drug delivery systems (SDDS) is notably to release or accelerate the release of the active ingredient in the right place, at the right time. It is essential in the development of such systems to study the release of the encapsulated active ingredient in the presence or the absence of appropriate stimulus. When it is possible, results obtained from smart sensitive systems are compared to those obtained with non-sensitive equivalent systems [53,77,129].

To study the stimulus-triggered drug release, in vitro experiments are performed. SDDS are incubated in conditions mimicking the stimulus (specific pH, temperature, redox potential). The release of the AI is then analyzed by qualitative methods to demonstrate the responsiveness to the stimulus or quantitative methods to establish the release kinetics of the AI.

#### 4.2.1. Proof of Concept Using Qualitative Methods

The Electron Paramagnetic Resonance (EPR) technique makes it possible to study paramagnetic molecules or materials (e.g., unpaired electrons) and to obtain valuable information on their structure and chemical environment. It is a powerful, non-invasive spectroscopic tool that can be used to monitor spin-labelled drug release processes in vitro and in vivo [130]. The EPR line shape displays the dynamic motions of the spin-labelled drugs, and a specific signal will be obtained for immobilized (i.e., encapsulated) or mobile drugs. Dong and co-workers used this method to investigate pH-triggered drug release. To use the EPR method, they loaded spin-labelled dexamethasone (DxPCA) into pH-sensitive Eudragit^®^ L100 nanoparticles (NPs). First, the NPs dispersion was diluted with pH 7.2 phosphate-buffered saline or pH 4 HCl solution. Then, EPR measurements were performed, and results showed that, with increase of the pH, the signal associated with mobile drugs increased, while the signal associated with immobilized (encapsulated) drugs declined in intensity [63].

#### 4.2.2. Release Kinetics Studies

Most of the time, the release of the drug is evaluated by putting the SDDS in contact with a liquid medium mimicking the stimulus. The released AI is then determined in the release medium with the appropriate analytical method, and a release kinetics curve can be drawn. In some rare cases, the SDDS, specifically hydrogels, do not hinder the analysis of the release medium. The hydrogel can be plunged in release media of different pH. The release medium is taken at a constant time for AI determination [44,45,46,48,61]. For example, Park and co-workers used this method to investigate the release efficiency of their pH-sensitive anionic gel. They examined the drug release behavior from this gel at pH 5.5 (the normal skin pH), 7.5 (the acne skin pH), and 8.5 (the atopic skin pH). The cumulative release of drug at pH 5.5, 7.5, and 8.5 after 24 h was 42%, 70%, and 73%, respectively [48], showing the pH responsiveness of the SDDS.

**Figure 5:**
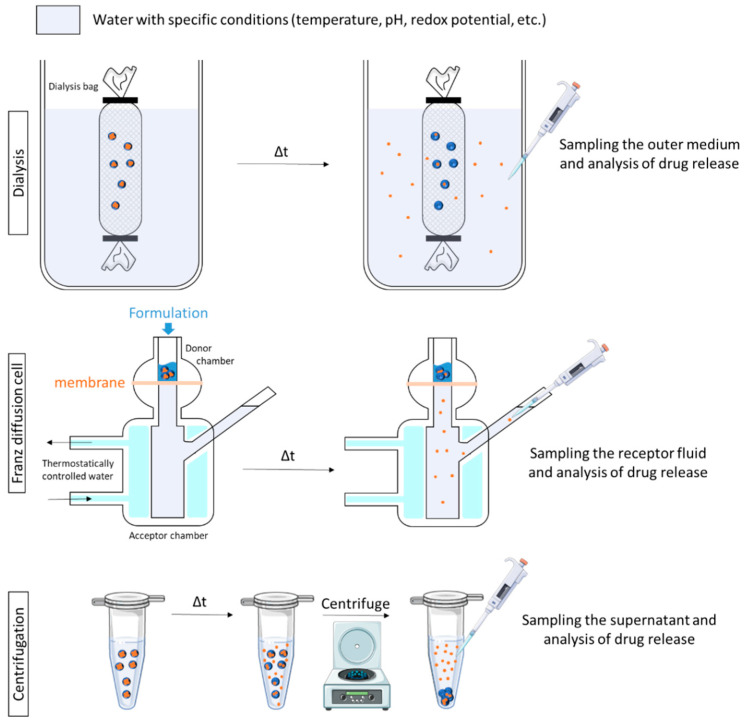
In vitro triggered drug release studies: methods used to separate the released drug from its smart system.

Most of the time, the release medium has to be separated from the SDDS to be analyzed. Three major methods of separation are described: the dialysis method, the use of Franz cells and centrifugation (Figure 5).

**Dialysis method.** Of all the methods used to assess drug release, the dialysis method is the most versatile and popular. In this method, physical separation of SDDS and the released drug is achieved using a dialysis membrane. Of the variety of dialysis method set-ups used, the most commonly cited is the dialysis bag [36,37,38,39]. Briefly, the SDDS is introduced into a dialysis bag of appropriate pore size, containing release media (inner media). It is subsequently sealed and placed in a larger vessel containing release media (outer media). At a scheduled time, an appropriate volume of the outer medium is withdrawn, and release kinetics can be established. This method is appropriate for pH triggering as the outer medium can be constituted of buffers showing different pH. Sahu and co-workers used the dialysis method to investigate the in-vitro drug release pattern of prepared pH-responsive cationic nanogels. They compared in vitro drug release results after using different saline phosphate buffer with different pH (4–7), and a significant drug release was observed at pH 4 [36].

**Franz-type diffusion cells.** Franz-type diffusion cells are used to study drug release with a synthetic membrane [49,56,65,73,101]. Franz cells consist of two primary chambers (donor and acceptor) separated by a membrane. The test product is applied to the membrane via the top chamber-donor compartment. The bottom chamber-acceptor compartment contains fluid from which samples are taken at regular intervals for analysis. For pH-triggered drug release studies, the acceptor compartment is filled with the buffer media of interest (specific pH). For temperature-triggered drug release, the system is kept in a temperature-controlled water bath to maintain the temperature of interest in the donor compartment. For example, Rozman and co-workers used this technique and maintained the temperature in the donor compartment at 32 °C or 20 °C in order to investigate vitamin release from temperature-sensitive microemulsion gel. Results showed that at 32 °C, 75% of vitamin C was released in 6h compared with 30% at 20 °C [73].

**Centrifugation.** More rarely, centrifugation is used to study drug release from nanogels. SDDS are placed in a medium with specific conditions (pH, temperature) and, at predetermined intervals, aliquots are withdrawn and centrifuged, and the supernatant is analyzed [34,35]. In other studies, SDDS are placed in a centrifugal filtering device and incubated at specific conditions. At certain time intervals, the samples are centrifuged, and the filtrate is analyzed [74,113].

### 4.3. Triggered Skin Penetration Studies

In in vitro stimulus-triggered drug release studies, there are two main types of techniques for skin penetration studies. Quantitative techniques include the use of diffusion cells. Qualitative or quantitative techniques are different microscopic and spectroscopic methods and the combinations thereof.

#### 4.3.1. Types of Skin Models

Penetration studies can be performed on artificial (Strat-M^®^ membrane [104]), human (reconstructed or excised skin [95]) or animal skin (usually rodents [75]). Even if most of the studies are performed on normal skin models, some authors develop specific skin models to get closer to the real use of the SDDS. For example, to mimic an impaired skin barrier, the skin can be tape-stripped 30 or 50 times [10,63,76,131]. To mimic atopic dermatitis (AD), filaggrin-deficient (FLG-) skin equivalent can be generated. The characteristics of those pathological skin models were highlighted by histology techniques, changes in the expression of epidermal barrier proteins and defects in the cutaneous innate immune response [74,76]. To mimic a chronic wound, a wound could be induced in normal human skin constructs by cutting the epidermal layer with a scalpel. To simulate the pH conditions of chronic wounds, wounded human skin constructs can be supplied with alkaline cell culture medium (pH 8) [54].

#### 4.3.2. Mapping the Active Ingredient 2D/3D Distribution in Skin

Microscopic techniques give important information about the spatial distribution of the active ingredient inside different skin layers or explain the mechanism of penetration.

Fluorescence microscopy is a frequently used method. Briefly, fluorescently labelled SDDS are applied to skin before incubation under specific triggering conditions. After incubation, skin (cryo)sections are observed under a fluorescence microscope, and pictures are taken and analyzed using the appropriate software. The mean fluorescence intensity of areas in different skin layers is then measured [10,77]. To visualize the effects of temperature on skin penetration and drug release, the temperature during incubation time can be modified. For example, in Asadian-Birjand’s study, labelled-nanogels were applied to human skin, and the skin sample was incubated at 4 °C or 37 °C for 4 h. Results showed higher fluorescence intensities in viable skin for samples incubated at 37 °C in comparison with the same nanogels incubated at 4 °C [77]. The release of the AI can also be triggered by an external stimulus. In Rancan’s study, skin samples with topically applied tagged tNG were irradiated for 30 s with an infrared (IR)-lamp. During this short time of irradiation, the skin surface temperature reached a maximum of 40 °C, as measured by means of an IR thermometer. The tNG then stayed in contact with the skin for two hours before analysis by fluorescence microscopy. The results showed enhanced penetration of tNGs with a release of dye in the epidermis upon thermal trigger [10].

Fluorescence measurements could be performed by means of a confocal laser microscope. This method gives the possibility to image deep into a three-dimensional biological sample like skin, at high resolution, high speed, in in vitro and in vivo conditions. Osorio-Blanco and co-workers used this technique in order to investigate the interactions and effects of thermoresponsive nanocarriers (NCs) on the stratum corneum of excised human skin. Briefly, rhodamine B was coupled to NCs before their application on excised human skin. Sample irradiation was performed for 30 s (using a standard IR lamp) to increase the skin surface temperature at 40 ± 2 °C. The samples were then incubated for 1000 min at a temperature below the phase transition point of the NCs. After incubation, skin sections were cut and analyzed by employing fluorescence microscopy imaging. The NCs-treated samples (with or without an IR trigger) were found to have a similar average fluorescence intensity in both upper and lower regions of the SC. However, thanks to stimulated Raman scattering (SRS) microscopy, which notably enabled them to trace deuterated water within the different layers of the skin, they showed that the thermoresponsive features of the NCs further enhanced skin hydration [95].

Fourier-transform infrared spectroscopy (FTIR) imaging can be performed to determine the distribution of the drug throughout the depth of the skin. Kang and co-workers used this technique in order to attest the interest of their thermosensitive solid lipid nanoparticles (SLNs) for improved dermal distribution. Briefly, the ex vivo skin penetration test of thermosensitive SLNs (or reference products) was conducted by using excised rat dorsal skin samples, mounted in the Franz-diffusion cell (37 °C). After 24 h, the skin was cut vertically into slices to determine the difference in drugs by depth. The skin slice was placed on a glass slide and examined by FT-IR imaging. The wavelength of 3450 cm^−1^, corresponding to a strong infrared absorption of the drug, was used for analysis. Results showed that smart SLNs delivered more drugs to deeper skin layers than the reference product [75].

#### 4.3.3. Quantitative Techniques

Franz-type diffusion (FD) cells and analogues are commonly used to study the skin penetration of AI. The principle is the same as that described above for release kinetics studies (part 4.2.2.), but instead of using synthetic membranes, reconstructed, human or animal skin are used. Different scenarios are then possible, which can be compatible with each other:

1. Samples of receptor fluid are taken at regular intervals and replaced with an equal volume of fresh medium. The samples are then analyzed by appropriate techniques (e.g., HPLC) for AI determination. The cumulative amounts of the drug are then plotted against the time, showing the permeation behavior [34,73,81,96].
2. At a specific time, at the end of the permeation study or before, the skin is removed from the FD cells, and the stratum corneum is wiped clean. The amount of AI retained in the entire skin or in each layer is determined after appropriate extraction [79]. The skin can be sectioned using different methods (e.g., cryo-sections [34,37,76], heating [73], forceps [80], or go through tape-stripping [46,48,75]) before AI determination in each layer.

To mimic the stimulus, which is the temperature gradient within the skin, the temperature of the setup was increased from 32 to 37 °C during skin penetration studies [74,76]. To mimic a release triggered by a heating source such as heat pad, Indulekha and co-workers used FD cells at two different temperatures. The first temperature, 32 °C, corresponds to normal skin temperature and the second one, 39 °C, corresponds to higher skin temperature, simulating the application of a heat pad. The temperature of 39 °C was maintained by shining the IR lamp on the skin with optimized time and exposure. Results showed that the cumulative AI permeated in the receiver compartment was significantly higher at 39 °C [103]. To mimic the temperature difference between the formulation (usually room temperature) and the skin surface (32 °C), Zavgorodnya and co-workers maintained the diffusion cell at 32 or 25 °C [104]. To mimic the pH difference between healthy skin (pH ~5) and diseased skin (pH ~7), Rizi and co-workers used FD cells with two different receptor media: one adjusted to pH 5 and one to pH 7. Results showed that all the formulations developed delivered significantly greater amounts of AI through porcine skin at pH 7 than at pH 5. Their approach demonstrates that it is feasible to deliver the active ingredient in diseased skin sites, where the pH is more alkaline than that of healthy skin [65].

### 4.4. In Vivo Efficacy

Some pathologies cannot be simulated in vitro. It is then necessary to evaluate the efficacy of the SDDS in vivo. For example, rodent models of inflammation, burns or wounds are used to assess the efficacy of SDDS [47,53,57]. Briefly, rodents are anesthetized, and their back is shaved and cleaned before being exposed to hot circular cylindrical devices [47,105] or excision [51,53]. After receiving the topical treatment of interest, representative photographs of wounds on animals and healing evolution are performed. After treatment period, rodents are sacrificed, and the skin of the back is surgically excised to be analyzed. For example, Mavuso et al. designed a topical delivery system with pH/redox responsive properties, which was supposed to have the ability to respond directly to the imbalance caused by skin inflammation. To prove the pH/redox responsiveness, two groups of rats were compared: a control group and a group with induced skin inflammation. Each group underwent administration of the relevant formulation, followed by blood sampling to evaluate the plasmatic AI concentration. Results displayed a higher degree of AI resorption in rats suffering from inflammation compared with the control group [57]. In another study, the in vivo efficacy of pH-responsive hydrogels was evaluated in a full thickness murine excisional wound model. Photographs of the wounds were taken weekly. At predetermined time, wound biopsies were collected for further histological and immunohistochemical study. Results revealed that a growth-factor-loaded wound-pH-responsive hydrogel induces a better healing response in comparison with a wound-healing-insensitive growth factor delivery system [53].

Jung and co-workers generated a psoriasis rat model. The hair was removed from the rat’s backs, after which 10% sodium dodecyl sulfate (SDS) in distilled water was applied for 2 days to generate a psoriasis model. Stratum corneum is known to be dried by SDS, which results in the layer becoming dehydrated and the composition of lipids in the layer changing. On day 3, the rats’ backs were divided into four parts and treated with different products including the SDDS of interest. Next, the back skin of the rats was excised on day 5 and 7 after treatment, and recovery of psoriatic skin was confirmed by hematoxylin and eosin staining [40].

## 5. Discussion

The literature on smart drug delivery systems (SDDS) increases exponentially and the variety of stimuli-responsive materials already described is significant. Analysis of the recent literature shows that interest in polymer-based SDDS is now present in the fields of dermatology and cosmetology, with the SDDS showing promising results in transdermal delivery.

Two major forms are developed: polymer-based hydrogels and nanosystems, particularly nanogels. Concerning hydrogels, their responsiveness to stimulus can be used for two main purposes: in situ gel formation or AI controlled release. In situ gel formation can be very interesting in the case of wound healing: the solution is applied to the wound, where it turns to a gel thanks to a specific pH or temperature of the skin. The liquid presentation allows users to tightly fit the contours of the wound, which ensures adhesion to the whole surface, while gelation ensures protection and constant moisturization of the wound. Swelling gels can also absorb wound exudates. Contrary to hydrogels, nanosystems can penetrate deeply into the tissue, according to their composition and surface properties [132,133,134], for example through hair follicles, and treat pathologies at deeper layers of the skin. Concerning the polymers used to prepare SDDS intended for skin application, their properties are selected to correspond to healthy or diseased skin. For temperature responsive systems, the release of the AI is triggered by the difference in temperature between the formula and the skin (immediate release) or by the gradient of temperature in the skin. Another possibility is to use an external device, like an infrared lamp, to trigger the release. It is not yet widely used as there is a risk of burning the lighted area. Nevertheless, this concept needs to be pursued as it could be a way for more specific delivery, and it could open the field to other polymers with higher VPPT and limit interindividual variability.

The majority of the SDDS intended for skin application are responsive to pH or temperature. They are linked to the specific physiology of the skin, leading to pH or temperature gradients in the normal or diseased skin. Nevertheless, the applications are limited to those stimuli, and researchers have to consider a wider diversity of stimuli that could be interesting in the dermatology or cosmetology fields, like the redox potential of skin cells, which varies in many skin conditions or pathologies, or take advantage of the enzymatic machinery present in the skin. In the same vein, only a few studies explore the use of external devices to trigger the SDDS.

Although interest in using SDDS for skin application has already been demonstrated in the literature, for the moment, only a few of those systems have been marketed [72]. This slow incorporation of smart nanocarriers in the market could be explained by several factors:

- Most materials are synthesized under poorly reproducible conditions, and the methods to prepare smart SDDS are not standardized.
- The developed polymer-based SDDS are considered to be ‘‘new excipients’’, and thus toxicity, biocompatibility and biodegradability are major issues that take time to be elucidated. That is why, in particular, alternatives to thermoresponsive NIPAM, which is not biodegradable, are studied, like ethylene glycol methacrylate or N-vinylcaprolactam. The use of natural polymers is also an interesting way to tackle the issue. Some authors started to slightly modify natural polymers to make them responsive to pH or temperature. This shift towards naturalness is all the more marked in the field of cosmetics.
- Another important point is the cost of production and evaluation of smart products, compared with already established dermocosmetics products. This is not discussed in the literature yet but has an important impact on the industrial feasibility of the systems. Our recent results indicated that the SDDS made of only 33% of the redox responsive mPEG-SS-PLA polymers were active in vitro [124]. Thus, the use of a mixture of stimuli-responsive and neutral polymers could be a way to control the SDDS cost.
- Different from traditional dosage forms, the impact of the stimulus-responsiveness on the release kinetics or efficacy of the SDDS has to be attested; therefore, there is a need for developing suitable analytical techniques.

This last point remains the most essential issue. Indeed, the fact that a smart polymer is used does not directly confer stimuli-responsiveness to a delivery system. One could expect a harmonization of the methods used to prove the implication of the stimulus in the efficacy of the systems. More than a simple demonstration of the sensitivity of the polymer, various available protocols and methods have to be applied in a complementary manner in order to have a complete vision of SDDS performance/limitations. In vitro release studies are very important to provide proof of concept. However, the release media often used are hydrophilic buffers that do not simulate the highly lipophilic skin surface. Moreover, it has been reported that the pH of the buffer influences the volume phase transition temperature (VPTT) of thermoresponsive polymers [53,135]. As the pH of the skin is acidic, it would be relevant to determine the VPTT value of a thermoresponsive system under conditions similar to those found on the skin. However, in studies of thermoresponsive systems for topical applications, very few take into account the acidic pH of the skin to evaluate the VPTT [53].

Due to the limits of in vitro attesting of the response to the stimulus, proof-of-concept studies of new SDDS should be completed with ex vivo experiments. Permeation tests are notably essential to measure the performance of smart transdermal delivery systems. Generally, human skin biopsies are the gold standard. However, the limited availability of fresh human skin leads to the use of defrosted skin. However, the latter is known for showing a low barrier function compared with human skin in vivo [136]. In the case of SDDS, it would be important to demonstrate that both the pH, redox potential and enzymatic activity are comparable to the skin in vivo. Animal skin is less and less used for permeation studies as reglementary and ethical issues render these models less attractive. In the last three decades, enormous efforts have been put into developing artificial membranes and cultured 3D models of human skin [137]. In the years to come, reconstructed skin could become a very interesting for testing SDDS as it shows more and more functionalities, including enzymatic activity [138]. Moreover, some reconstructed models of skin disease like atopic dermatitis have been developed recently [139].

Some skin diseases or conditions cannot be reproduced in vitro or ex vivo. It is then necessary to test SDDS on animal models. For certain applications, the stimulus is generated by the skin (inflammation, wounds). In this case, the lesion must be well characterized to demonstrate that the local pH or temperature is comparable in both reconstructed and natural human skin. For other applications, like psoriasis or atopic dermatitis, the stimulus has to be artificially generated by chemicals. This field could benefit from the harmonization of practices to allow for comparison of the efficacy of the systems.

## 6. Conclusions

The skin is a key site for local and systemic drug delivery. It has the unique qualities of being easily accessible yet relatively impermeable. Overcoming the remarkable skin barrier properties in an efficient, temporary and safe manner remains a challenge. To enhance their efficacy and reduce the related side effects, active ingredients should selectively accumulate in the disease area with high controllability. Smart drug delivery systems (SDDS) sensitive to a specific environment can be part of the pharmaceutical arsenal to treat dermatological pathologies. In this field, smart polymers are mostly used to develop SDDS, such as polymer-based nanogels or hydrogels.

Scientists have innovated the field of the dermo-cosmetic formulations, as well as the analytical methods (in vitro, ex vivo or in vivo) used to attest to their interest in increasing the efficiency of products. Many SDDS show a better efficacy than classic dosage form. Even if the most commonly used stimuli remain the pH of the skin (healthy and/or diseases) and the temperature (surface or gradient), other stimuli, such as the redox potential, light, enzymes, electric fields, etc., are increasingly studied to trigger the release of active ingredients in the dermatological and cosmetic fields, which will undoubtedly make it possible to improve the management of skin pathologies. Likewise, significant progress is expected in the developments of smart transdermal drug delivery systems.

## Figures and Tables

**Table 1:** Structure of pH-responsive polymers used to design smart drug delivery systems for skin application.

pH-Sensitive Polymers		Structure of the Monomer	Type of SDDS	Application	Ref.
**Cationic System**	Chitosan (≅ chitin with % degree of de-acetylation)	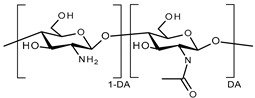 DA: degree of acetylation	Nanosystems	Topical therapy of skin cancers	[34,35,36,37,38,39]
				Treatment for atopic dermatitis	[40]
			Hydrogels	Textile-based transdermal therapy	[41]
				Wound dressings	[42]
	Dimethylaminoethyl-functional methacrylate Eudragit E100	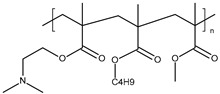	Microsystems	Skin care	[43]
**Anionic System**	Carboxymethyl Chitosan	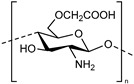	Hydrogels	Transdermal drug delivery system	[44]
	Hyaluronic acid (HA)	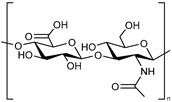	Hydrogels	Transdermal delivery systems for skin lesions	[45]
				Transdermal delivery system for psoriasis skin relief	[46]
				Textile-based transdermal therapy	[41]
				Wound healing to treat skin burn lesions	[47]
	Carboxymethyl cellulose (CMC)	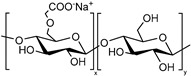	Hydrogels	Potential treatment of atopic dermatitis	[48]
	Cellulose acetate phthalate	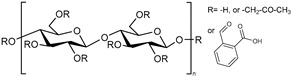	Nanosystems	Dermal carriers	[49]
	Hydroxypropyl methyl cellulose phthalate (HPMCP)	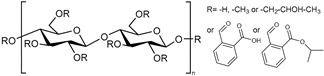	Nanosystems	Dermal carriers	[49]
	Carboxylated agarose	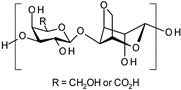	Hydrogels	Wound dressings	[50]
	Alginate	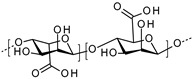	Microsystems	Wound healing	[51]
	Keratin	-	Hydrogels	Antimicrobial wound dressing	[52]
**Anionic System**	Poly(acrylic acid)	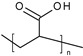	Hydrogels	Wound healing	[53]
				Treatment of chronic wounds	[54]
				Antibacterial wound dressing application	[55]
	Poly(methacrylic acid)	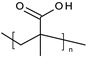	Nanosystems	Dermal carriers	[49,56]
				Transdermal delivery system	[57]
				Delivering cosmetic agents to melanocytes	[58]
			Microgel	(Trans)dermal drug delivery system	[59]
			Hydrogel	Smart delivery system for cosmetic ingredients	[60]
	Poly(maleic acid)	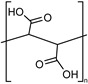	Hydrogels	Local therapeutic transdermal delivery applications	[61]
	Poly(itaconic acid)	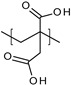	Hydrogels	Treatment of bacterial infections	[62]

**Table 2:** Structure and characteristics of thermoresponsive polymers used to design smart drug delivery systems for skin application.

Thermoresponsive Polymers	Monomer Structure	Type of SDDS	T°C Phase Transition	Application	Ref.
Poloxamers (or Pluronics^®^)	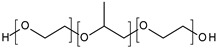	Nanosystems	~25 °C	Treatment of cutaneous Leishmanias.	[79]
			-	Delivery drugs into deep skin layers	[75]
		Hydrogels	-	Skin delivery against hypertension	[88]
			32 °C	Dermal delivery system	[81]
			37 °C	Atopic dermatitis treatment	[89,90]
			~20 °C	Topical formulations	[91]
			37 °C	Skin inflammation and wound healing	[92]
			36.7 °C	Wound healing application	[93]
			~24°C or 30.4 °C	Topical therapeutic formulation	[94]
			32 °C	Wound healing to treat skin burn lesions	[95]
			30 °C	Textile-based transdermal therapy	[41]
Poly(*N*-isopropylacrylamide)(PNIPAM)	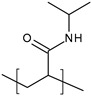	Nanosystems	31 to 37 °C	Topical drug delivery carrier	[96]
			35 °C	Topical delivery systems for biomacromolecules	[76]
			~33 °C	Dermal delivery	[97]
			~34 °C	Cutaneous drug delivery	[98]
			41.2 °C	Topical delivery systems	[87]
			~41 °C	Skin penetration enhancer	[95]
		Microsystems	30–34 °C	Develop a novel unique wound dressing	[99]
			34 °C	Transdermal delivery systems	[100]
		Hydrogels	35–36 °C	Wound healing	[53]
			~32 °C	Treatment for psoriasis skin relief	[46]
			36 °C	Topical administration (vaginal drug delivery)	[101]
Poly(*N*-isopropylmethacryl amide) (PNIPMAM)	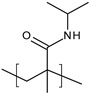	Nanosystems	34 °C	Skin penetration enhancer	[95]
Poly(ethyl glycidyl ether-co-methyl glycidyl ether)	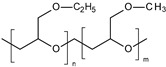	Nanosystems	34 °C	Treatment of severe skin diseases	[98]
			~34 °C	Treatment of inflammatory skin diseases	[10]
			~32 °C	Topical delivery into barrier-deficient skin	[74]
Poly(ethylene glycol) methacrylate	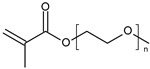	Nanosystems	36 °C	Applications in dermatotherapy and transdermal drug delivery	[77]
			>35 °C	Delivering cosmetic agents to melanocytes	[58]
oligo(-ethylene glycol)-decorated polyisocyanopeptide (PIC)	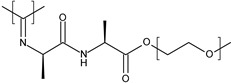	Hydrogels	RT	Facilitate wound repair	[102]
Poly(*N*-vinyl caprolactam) (PVCL)	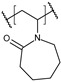	Hydrogels	35 °C	Transdermal drug delivery system for pain management	[103]
			~32 °C	Develop skin-sensitive materials for topical drug delivery	[104]
PEG-PCL-PEG PEG: Poly(ethylene glycol) PCL: Poly(e-caprolactone)	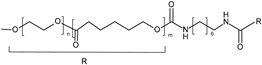	Nanosystems	~37 °C	Improve liposomes adhesion for wound healing	[105]
		Hydrogels	34 °C	Applications in skin care and wound treatment	[106]

## Data Availability

Not applicable.
